# Al and Cu Effect on the Microstructure and Mechanical Properties of HEA Based on the AlCoCuFeNi System

**DOI:** 10.3390/ma18194564

**Published:** 2025-09-30

**Authors:** Konrad Chrzan, Barbara Kalandyk, Małgorzata Grudzień-Rakoczy, Łukasz Rakoczy, Kamil Cichocki, Robert Żuczek, Filip Kateusz, Aleksandra Bętkowska, Adelajda Polkowska, Justyna Kasińska

**Affiliations:** 1 Łukasiewicz Research Network, Krakow Institute of Technology, 73 Zakopiańska, 30-418 Krakow, Poland; malgorzata.grudzien@kit.lukasiewicz.gov.pl (M.G.-R.); robert.zuczek@kit.lukasiewicz.gov.pl (R.Ż.); filip.kateusz@kit.lukasiewicz.gov.pl (F.K.); aleksandra.betkowska@kit.lukasiewicz.gov.pl (A.B.); adelajda.polkowska@kit.lukasiewicz.gov.pl (A.P.); 2 Faculty of Foundry Engineering, AGH University of Krakow, 30 Mickiewicza Ave., 30-059 Krakow, Poland; bk@agh.edu.pl; 3 Faculty of Metals Engineering and Industrial Computer Science, AGH University of Krakow, 30 Mickiewicza Ave., 30-059 Krakow, Poland; lrakoczy@agh.edu.pl (Ł.R.); cichocki@agh.edu.pl (K.C.); 4 Faculty of Mechatronics and Mechanical Engineering, Kielce University of Technology, 7 Tysiąclecia Państwa Polskiego Ave., 25-314 Kielce, Poland; kasinska@tu.kielce.pl

**Keywords:** HEA, AlCoCuFeNi, microstructure, mechanical properties

## Abstract

Three variants of high-entropy alloys (HEAs) from the AlCoCuFeNi group, containing different amounts of Al and Cu, were developed and produced via induction melting and casting into ceramic moulds. The ingots were homogenized at 1000 °C for 10 h. Analyses revealed that variations in Al and Cu concentrations led to significant changes in the material’s microstructure, hardness, strength, and impact strength. In the equiatomic variant, differential scanning calorimetry revealed a peak associated with the phase transformation, indicating that this alloy’s microstructure consists of two distinct phases. In contrast, when the concentrations of Al and Cu are reduced, a single-phase microstructure is observed. The equiatomic variant (used as a reference) is characterized by its hardness and brittleness, exhibiting slight ductility, with a tensile strength of 80 MPa, a hardness of 400 HV5, and an impact strength of 1.9 J/cm^2^. However, with adjusted Al contents of 1/2 and Cu contents of 1/4, the alloy displays exceptional strength combined with good plasticity, achieving a tensile strength of up to 450 MPa with 60% elongation, and an impact strength of 215 J/cm^2^. The non-equiatomic variants exhibit a comparatively more straightforward microstructure and enhanced ductility, which may facilitate easier processing of these alloys. Fractography investigation revealed a ductile mode of fracture in the samples.

## 1. Introduction

High-entropy alloys (HEAs) represent an innovative and rapidly evolving category of engineering materials. Their unique properties allow them to compete with expensive Ni-based and Co-based superalloys currently in use. HEAs are classified as multicomponent alloys, a development that began in 2004 by Yeh [1] and Cantor [2]. A key feature of HEAs is the absence of a single dominant element in their composition; instead, the alloy’s elements are present in similar proportions [3]. To qualify as a HEA, an alloy must contain at least five different elements, with each element making up between 5% and 35% of the total atomic volume. Contrary to earlier assumptions, HEAs typically form a solid solution structure. This behavior is attributed to four characteristic effects: the high-entropy effect [4,5], strong crystal lattice distortion [6], slow diffusion [7], and the cocktail effect [8]. Consequently, many HEAs exhibit excellent properties that make them competitive with existing materials. Alloys with a face-centered cubic (FCC) structure are known for possessing good plasticity but lower strength [9]. A notable example is the Al_0.3_CoCrFeNi alloy, which can achieve around 60% elongation and a tensile strength of 300 MPa in its as-cast state at room temperature [10]. Additionally, alloys from the FeNiMnAlCr group demonstrate similar strength properties [11,12,13].

Alloys with a body-centred cubic (BCC) structure are known for their relatively high strength, accompanied by reduced ductility [14]. For example, alloys from the WNbMoTaV group [15,16] and those based on NbMoTaW elements [17,18] fall into this category. Furthermore, FeAlMnSiC-based high entropic steels exhibit fascinating properties, especially under cryogenic conditions, showing increased elongation and tensile strength [19,20]. The favourable ratio of tensile strength to ductility is often attributed to the TWIP and TRIP effects in these materials [21,22,23]. Owing to their excellent resistance to abrasion and compression, HEAs, such as those from the AlCoCrFeNi group [24,25], are utilized as functional coatings.

These materials are produced using various methods, such as Physical Vapor Deposition (PVD) [26,27] or powder metallurgy [28,29,30]. However, when the goal is to create a bulk material rather than a coating, the elements that comprise the alloy are typically remelted. Many studies describe the production process using smelting in a laboratory arc furnace [31,32,33]. This method offers the advantage of producing a small amount of material, which helps keep testing costs low. However, due to the numerous elements present in HEAs, materials melted this way often experience segregation, resulting in heterogeneous compositions [34,35]. To resolve this issue, experimental ingots are typically remelted multiple times and subjected to extended annealing to achieve better homogenization of the alloy [36]. An alternative approach involves using induction furnace for alloy melting. This method enhances the mixing of the liquid metal in the crucible, leading to improved homogeneity throughout the metal bath. Nonetheless, this process also typically requires remelting the material to further reduce segregation among the alloying elements [37]. Some drawbacks of induction melting include longer production times and the need for careful control of the atmosphere over the bath. It is essential to note that further research is needed to understand how induction melting impacts the uniformity of HEA alloys, partly due to the larger quantity of material required to initiate the melting process with this technology.

This research aims to demonstrate how melting technology using an induction furnace can influence the microstructure and specific properties of a HEAs from the AlCoCuFeNi group, which has been designed and manufactured. Three different variations of the alloy were produced, each containing varying amounts of Al and Cu to fulfill the criteria for HEAs. It is important to note that the specific variations of the alloy derived from the cast HEA AlCoCuFeNi group have not yet been tested. However, the combination of elements shows promise regarding its properties and potential applications.

## 2. Materials and Methods

Three variations of HEAs, comprising elements such as Al, Co, Cu, Fe, and Ni, were chosen for testing. The chemical composition of the AlCoCuFeNi alloys is presented in Table 1. The alloys designated D1 and D3 constitute newly developed materials for which no prior studies have been reported, apart from those conducted by the authors of the present investigation. Although the properties of equiatomic alloys have been addressed in the literature, the findings remain highly inconsistent, with the discrepancies largely governed by the processing route. The present study focuses on materials fabricated under conditions closely resembling industrial practice, specifically by means of induction melting in a ceramic crucible.

In the notation used for HEAs, the selected variants can be presented in the following form: D1—as Al_1/4_CoCu_1/4_FeNi, D3—as Al_1/2_CoCu_1/4_FeNi, and D5 as a equiatomic AlCoCuFeNi. Metals with a purity of at least 99.9% were used as input materials to melt in an induction furnace (Table 2).

The melting process was conducted in a laboratory induction furnace, the UltraMelt 4/5 (Ultraflex Power, Ronkonkoma, NY, USA), using a replaceable multilayer crucible with a capacity of 4 kg. During melting, an inert gas was introduced onto the surface of the molten metal. The liquid metal was then poured into ceramic molds. After the casting process, the castings were removed from the molds and underwent homogenizing annealing at 1000 °C for 10 h.

Metallographic samples were cut off from the casting ground with SiC sandpaper and polished using diamond suspensions (3 μm, 1 μm) and colloidal silica (0.06 μm). The samples were chemically etched in Adler reagent. Light microscopy (LM) observations were performed using a Leica MEF 4M microscope (Leica Camera AG, Wetzlar, Germany). Scanning electron microscopy observation has been carried out with FEI Inspect S50 equipped with an EDS detector (FEI Company, Hillsboro, OR, USA). The accelerating voltage during imaging and chemical composition measurements was equal to 30 keV. The surface fraction of phases was analyzed by a Widefield Metallographs instrument with Leica QWin software (V3.0), capturing 12 microstructure images.

Phase composition of samples (10 mm × 10 mm × 5 mm) was analyzed using a Panalytical Empyrean diffractometer equipped with a copper anode and a PIXcell 3D detector (Malvern Panalytical Ltd., Malvern, UK). Data analysis was conducted using PDF-5 + 2024 database. Measurements were carried out at room temperature in 2θ = 5–100° with step size of 0.0263.

To determine the phase transformations temperatures of materials differential scanning calorimetry using DSC 404C Pegasus device from Netzsch (Erich NETZSCH B.V. & Co. Holding KG, Selb, Germany) was performed. Each sample, weighing about 40 mg, was placed in alumina crucibles with a lid and heated to 1000 °C at a rate of 10 °C/min in an inert atmosphere (argon).

The hardness of the alloys was measured using the Vickers method on a Duramin-40M1 hardness device (Struers, Copenhagen, Denmark) with a load of 5 kgf. Additionally, the microhardness of the selected phases was assessed with a Nexus 4000 hardness tester (Innovatest) using a load of 0.1 kg (Struers A/S, Copenhagen, Denmark).

The strength at room temperature was determined through tensile tests conducted in accordance with the DIN EN ISO 6892-1/ISO 10113/ISO 10275 standard [38]. The testing was performed using a Zwick Roell Z010 machine, which was equipped with a laserXtens 1–15 HP laser extensometer (ZwickRoell GmbH & Co. KG, Ulm, Germany). Flat samples with a cross-section of 2 mm × 2 mm were used. Each variant of the HEA alloys underwent at least three tests.

Impact strength tests were conducted at room temperature using a Hoytom Charpy (KB Pruftechnik Gmbh, Hochdorf-Asseiheim, Germany) hammer with an energy capacity of 300 J. The tests used standard samples measuring 10 mm × 10 mm × 55 mm, each featuring a 2 mm deep “V” notch. After the impact tests, the surfaces of the alloys were analyzed with a Keyence VHX digital microscope (Keyence Corporation, Mechelen, Belgium). The 3D maps were generated from this analysis, and roughness parameters (R_a_ and R_z_) were subsequently calculated based on these maps. Fractures have been subjected to analysis by scanning electron microscopy.

## 3. Results

### 3.1. Microstructure of the Fabricated HEAs

**Figure 1:**
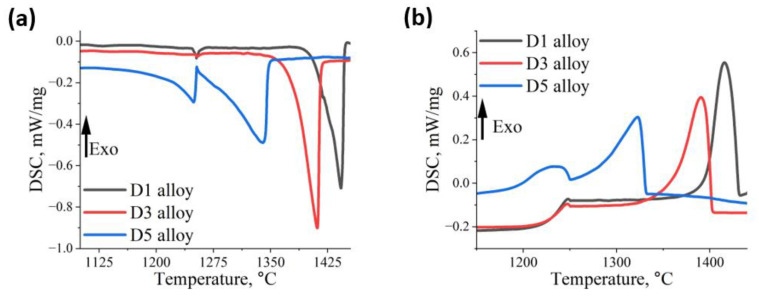
DSC curves of the investigated alloys registered during (**a**) heating and (**b**) cooling.

To analyze the thermal effects during heating and cooling of the HEAs, differential scanning calorimetry was performed (Figure 1, Table 3).

During the heating of D1 and D3 alloys, a fine endothermic effect was observed at 1253 °C. This effect can be attributed to the dissolution of very fine precipitates, e.g., intermetallic L1_2_ phase, which is influenced by the presence of Ni and Al in the chemical composition. In contrast, for D5 alloy, the endothermic effect is more pronounced and occurs at a slightly lower temperature of 1248 °C. This is likely linked to the dissolution of precipitates that display bright phase contrast, which will be discussed in more detail later in the article. The solidus temperatures for D1, D3, and D5 alloys are 1385 °C, 1335 °C, and 1129 °C, respectively. Correspondingly, the liquidus temperatures also show a decreasing trend, measured at 1442 °C for D1, 1411 °C for D3, and 1340 °C for D5. As a result, the solidification intervals for these alloys are 57 °C for D1, 76 °C for D3, and as wide as 211 °C for D5. The significant reduction in the solidus temperature of D5 alloy can be attributed to its higher content of low-melting-point elements such as Al and Cu, which broadens the solidification range. Analysis of the cooling curves confirmed the same correlations between phase transformation temperatures and chemical composition that were observed during heating. Solidification begins at 1415 °C, 1390 °C, and 1322 °C for alloys D1, D3, and D5, respectively, and ends at 1361 °C, 1317 °C, and 1187 °C. Additionally, in D5 alloy, a strong exothermic effect was detected at 1230 °C, suggesting that some of the strengthening phases may precipitate through a eutectic transformation.

**Figure 2:**
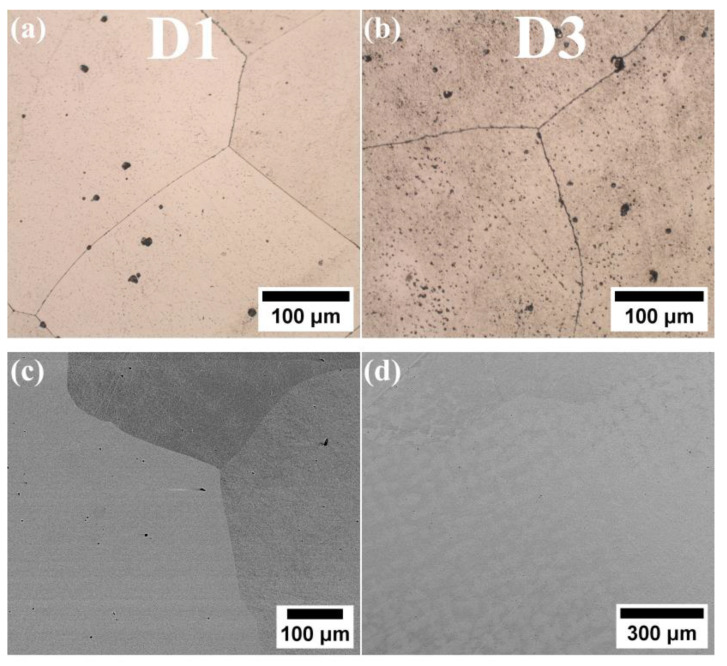
Microstructure of HEA: (**a**,**c**) D1; (**b**,**d**) D3.

**Figure 3:**
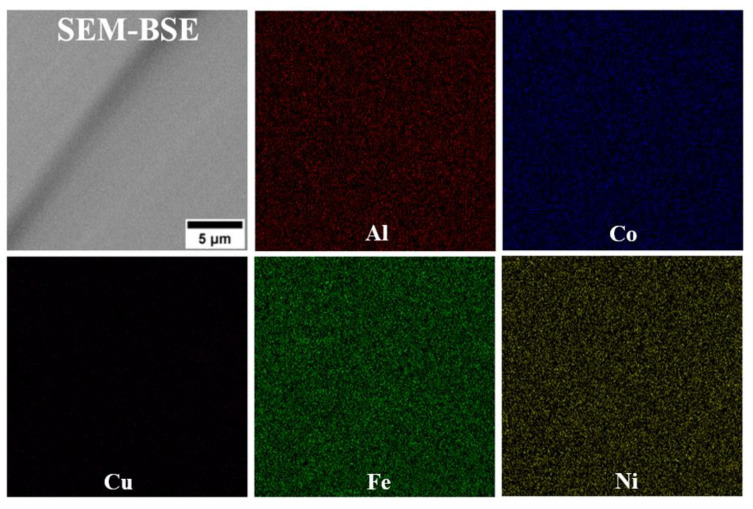
SEM-EDS mapping of selected alloying elements in D1 alloy.

**Figure 4:**
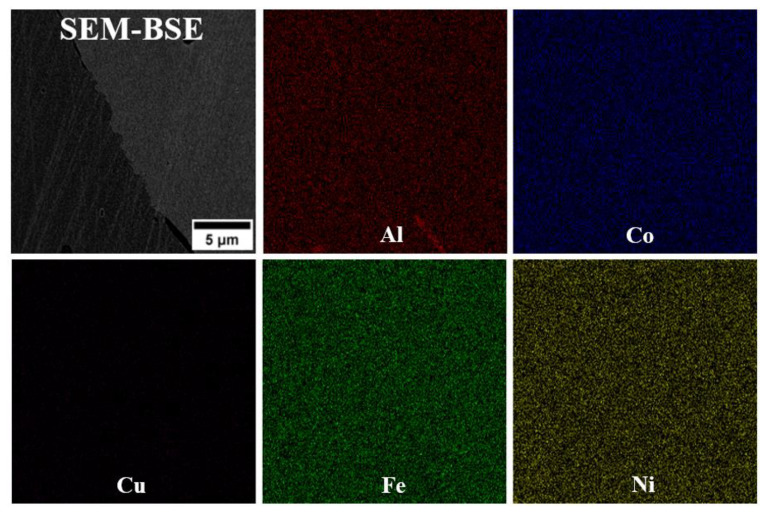
SEM-EDS mapping of selected alloying elements in D3 alloy.

**Figure 5:**
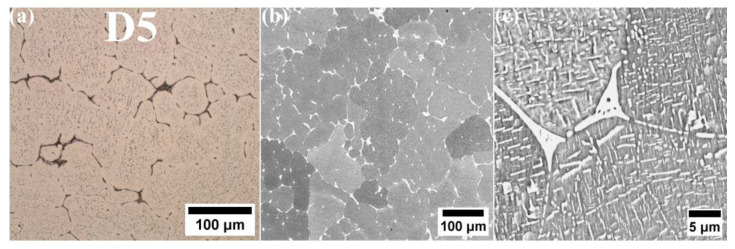
Distribution, amount and morphology of strengthening precipitates in D5 alloy. (**a**) light microscopy; (**b**) SEM image—200× magnification; (**c**) SEM image—1500× magnification.

**Figure 6:**
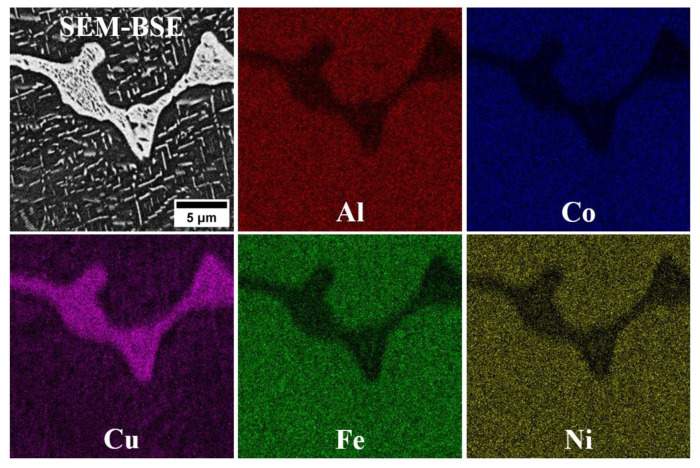
SEM-EDS mapping of selected alloying elements in D5 alloy.

The microstructure analysis of alloys D1 and D3 shows that, despite their differing chemical compositions, both exhibit a single-phase microstructure characterized by large grains (Figure 2). Lack of second-phase precipitates is observed at the grain boundaries, indicating a fully solid solution microstructure. SEM-EDS analysis reveals a uniform distribution of alloying elements in both D1 and D3 alloys, supporting the likelihood of a single-phase solid solution microstructure. The SEM-EDS mapping of both alloys indicated that all alloying elements are uniformly distributed throughout the microstructure (Figure 3 and Figure 4). While fine dark precipitates are present in both alloys, the results of SEM-EDS analysis suggests that they are likely fine slag inclusions from the melting and pouring processes. On the other hand, it could be a result of specimens etching. In contrast, the microstructure of alloy D5 (equiatomic variant), which contains 20% Al along with Cu, Co, Fe, and Ni, has undergone significant changes. A distinct phase with a different morphology is visible along the grain boundaries (Figure 5), and under higher magnification, fine plate-like precipitates are within the grains. The surface distribution of elements for the D5 alloy revealed that larger particles at the grain boundaries are rich in copper (Figure 6). The results of the SEM-EDS chemical composition analysis performed for this alloy are presented in Table 4. They indicate that this phase is also enriched in Fe and Co but depleted in Al (its content decreased from 20% to 12.3%). 

**Figure 7:**
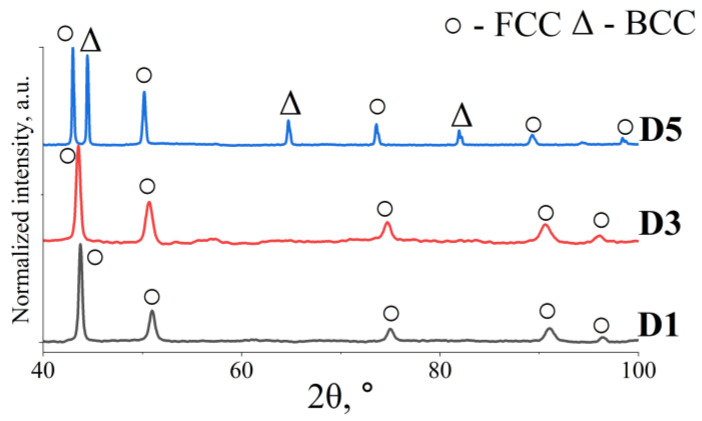
XRD spectra of the investigated HEA.

XRD analysis indicates that the materials labelled D1 and D3 are primarily single-phase alloys (Figure 7). In contrast, the diffraction pattern of D5 alloy suggests the existence of at least two distinct phases.

Changes in the concentrations of Al and Cu can lead to the formation of very fine precipitates, particularly along grain boundaries. This phenomenon is described in detail by Ren et al. [38]. The low concentration of these phases resulted in the X-ray diffraction (XRD) tests not revealing their presence.

### 3.2. Mechanical Properties of the High Entropy D1, D3 and D6 Alloys at Room Temperature

#### 3.2.1. Hardness and Tensile Test

The results indicate that alloy D1, which is rich in Co, Fe, and Ni, exhibited the lowest mean hardness among all tested variants, equal to 95 HV5. The D3 alloy, which is also enriched with Co, Fe, and Ni, contains nearly twice the amount of Al and slightly less Cu, resulting in a higher hardness of 133 HV5. In both alloys, additional precipitates in large amounts are not visible. Therefore, the increase in hardness is most likely influenced by the solution hardening mechanism. The grain sizes between these two variants are comparable. The two-phase D5 alloy demonstrated the highest hardness, measuring 403 HV5 [39]. The microstructure of the D5 alloy comprises two distinct phases. Microhardness measurements indicated that the phase exhibiting a bright contrast attained a microhardness of 761 HV0.01 (±8), which is almost three times greater than that of the matrix, measured at 281 HV0.01 (±15).

**Figure 8:**
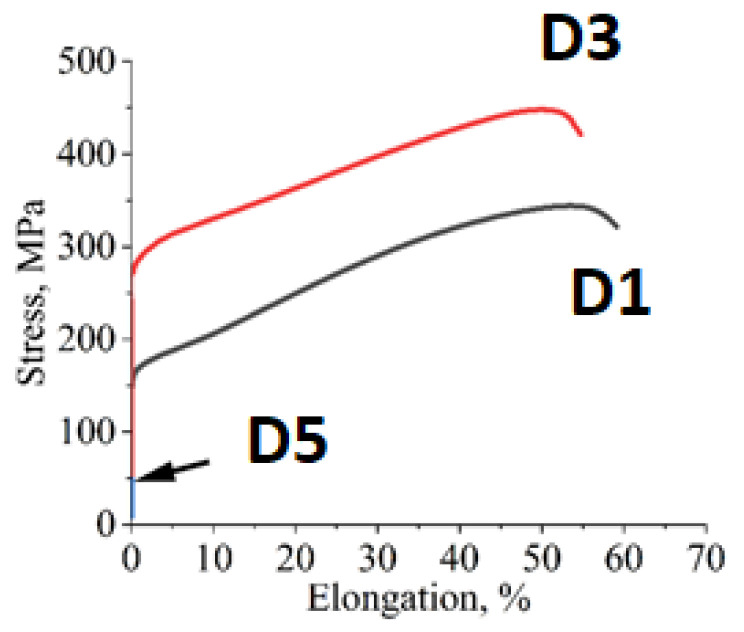
Tensile test curves of HEA: D1, D3, D5.

The results of the tensile tests are shown in Figure 8 and Table 5. Alloys D1 and D3 exhibited satisfactory tensile strengths of approximately 369 MPa and 465.7 MPa, respectively. Additionally, both alloys displayed significant ductility reserves, with values of approximately 50% for D1 and 61% for D3. The Young’s modulus (E) was measured at 183.5 GPa for alloy D1, while alloy D3 exhibited a lower value of 120.6 GPa. Based on the results, it was determined that alloy D3 exhibited the highest yield strength and tensile strength, as well as greater ductility compared to the other variants. In contrast to D1, D3 contains a higher Al content and slightly lower amounts of other elements (as shown in Table 1). On the other hand, alloy D5, which has a two-phase microstructure and equal content of alloying elements, was found to be hard (403 HV5) but very brittle. This alloy underwent minimal plastic deformation, and its mean strength was only 49 MPa, a value that was difficult to ascertain due to its excessive brittleness.

**Figure 9:**
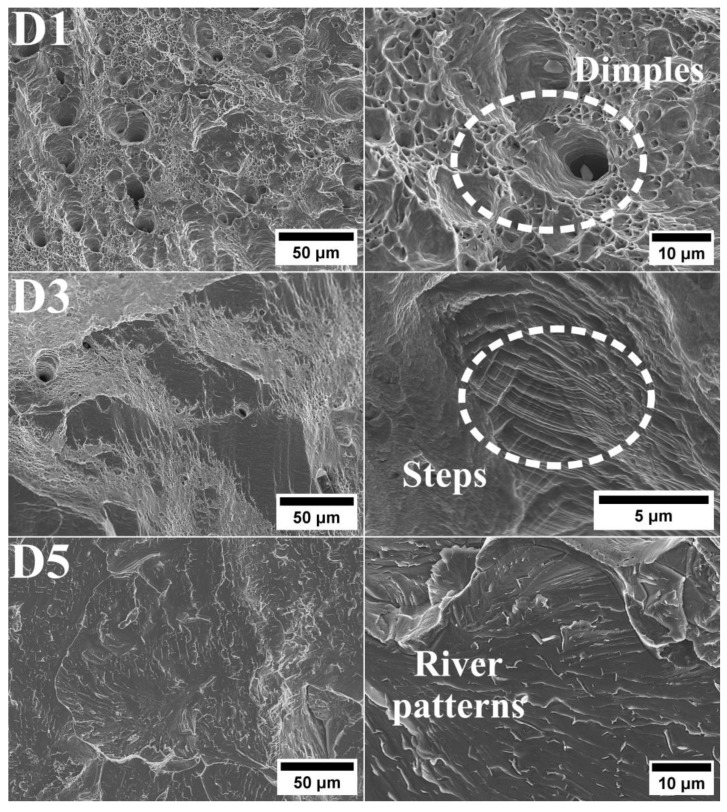
Fracture of the HEA after tensile test.

Alloy D5 contains numerous precipitates that are enriched in Cu. These precipitates are arranged in an orderly manner and are also found at grain boundaries. This alloy is characterized by very low tensile strength and virtually no plasticity, indicating that the precipitates significantly affect its strength and brittleness. Based on the results obtained, it can be concluded that alloys D1 and D3 are ductile, whereas alloy D5 should be classified as brittle. For alloys D1 and D3, these surfaces reveal a ductile fracture mechanism (Figure 9). Both alloys exhibit voids that have formed around precipitates, a characteristic feature of ductile fracture. The larger size of the voids observed on the fracture of D3 alloy indicates it has a greater capacity for plastic deformation compared to D1 alloy. These variations in fracture structure are consistent with the results obtained from uniaxial tensile tests on both variants. Additionally, fine inclusions of various shapes can be found within the voids. The significant depth of these voids highlights the high plasticity of the tested alloys. In some regions, fine non-metallic inclusions of diverse shapes are also visible. The fracture of alloy D5 exhibited negligible ductile deformation, accompanied by noticeable local deformations. Microscopic analysis revealed that the fracture of D5 alloy has a brittle transcrystalline nature, featuring numerous visible faults that form characteristic patterns resembling rivers and river basins. Additionally, microcracks and fine inclusions are observed. The varying patterns of these visible faults may result from grain boundaries with different orientations, which can inhibit the progression of a cleavage fracture.

#### 3.2.2. Impact Test

The results of the impact test conducted on the investigated alloys are summarized in Table 6. As anticipated, alloy D5, which had the highest hardness, displayed the lowest impact energy following the tensile test. The fracture of this alloy does not exhibit any signs of plastic deformation. In contrast, significant differences in cracking behavior were observed in the other alloys, specifically D1 and D3, when compared to D5. Notably, alloys D1 and D3 demonstrated considerable plastic deformation in the areas of cracking. Additionally, cracks were noted in regions away from the notch, suggesting significant strengthening of the material.

**Figure 10:**
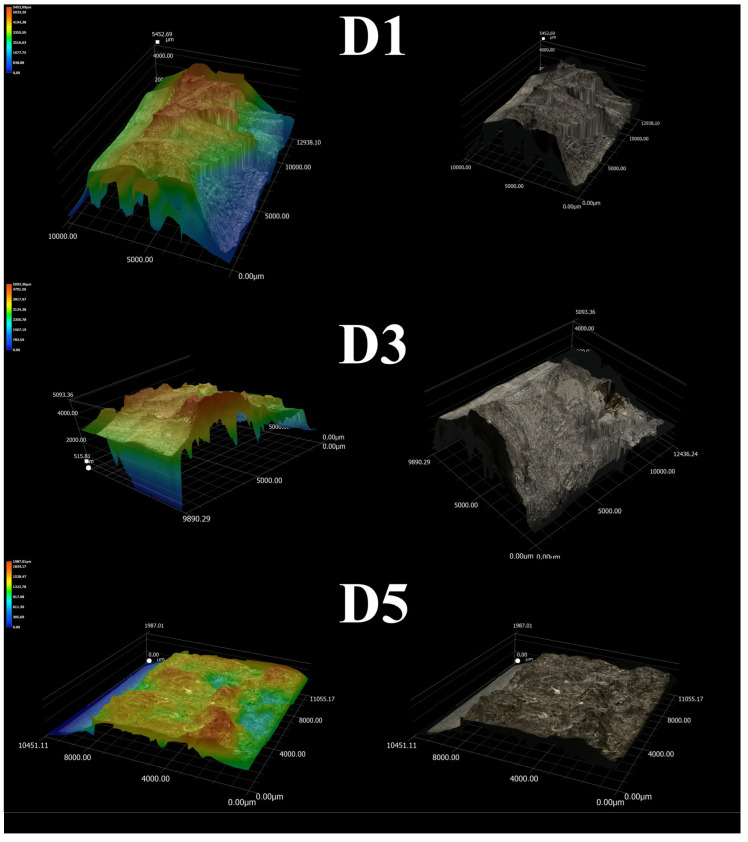
Differences in cracking mechanism presented on 3D surface of HEA after impact test.

Figure 10 shows representative surfaces of fractured samples after the Charpy impact test. The nature of plastic deformation suggests that alloys D1 and D3 exhibit higher ductility compared to the D5 alloy. The height of the profiles was measured on the post-test samples to highlight the differences in plastic deformation. Each specimen had six measurement lines generated: three vertical and three horizontal. The calculations excluded the area of the notches. The D1 and D3 alloys displayed a more irregular surface shape compared to the D5 alloy. The average roughness parameters were R_a_ = 715 µm and R_z_ = 2579 µm for the D1 alloy, and R_a_ = 458 µm and R_z_ = 1926 µm for D3 alloy. For D5 alloy, the R_a_ parameter was 139 µm, while R_z_ was 740 µm. The nature of the surface and the results indicate that D1 and D3 alloys experienced significant plastic deformation during fracture, characterized by numerous peaks. In contrast, the surface of the D5 alloy showed less plastic deformation, and the shape of its cross-section remained relatively unchanged.

## 4. Discussion

The tested alloys exhibit variations in both microstructure and mechanical properties. The concentrations of Al and Cu have a significant influence on these characteristics. In the D1 and D3 alloys, the microstructure appears predominantly single-phase, with no large precipitates visible. More advanced techniques, such as TEM, are recommended for an accurate characterization of these alloys. In contrast, D5 alloy contains clearly observable precipitates. XRD analysis confirmed the presence of at least two phases in D5 alloy. Alloys with reduced concentrations of both elements demonstrate considerably higher plasticity compared to the equiatomic D5 alloy. The differences between D1 and D3 alloys are minor, with only a slight increase in hardness and comparable tensile strength. The D5 alloy, however, exhibits very poor tensile performance in terms of both ductility and ultimate tensile strength. The results of the impact toughness tests highlight significant differences between the tested alloys, with alloy D5 exhibiting approximately ten times lower toughness compared to the others. Fractographic analysis shows distinct differences in the cracking mechanisms. The D1 and D3 alloys display regions that indicate plastic deformation, while D5 alloy has a noticeably different fracture topography.

## 5. Conclusions

- Variations in Al and Cu content significantly affect the microstructure and mechanical properties of the three HEA from the AlCoCuFeNi group.
- In the microstructure of the equiatomic D5 alloy, the two phases exhibit distinct chemical compositions and hardness. The SEM-EDS analysis indicates that Al and Cu tend to segregate significantly, whereas Co shows the least susceptibility to segregation.
- In D3 alloy, where the Al content increased to 13.33% while keeping the Cu content comparable to that of the D1 alloy, an enhancement in the mechanical properties was observed when compared to D1 (R_m_ = 466 MPa and R_p0.2_ = 221 MPa). This was achieved while also maintaining satisfactory elongation of 60.6%. However, it was noted that the impact energy of D3 alloy was 20 J lower than that of D1 alloy.
- The equimolar alloy D5 exhibited the lowest strength and impact energy.
- Fractographic analysis confirmed the brittle nature of the fracture in D5 alloy. In contrast, D1 and D3 alloys exhibited larger areas of ductile fractures characterized by prominent dimples.

## Figures and Tables

**Table 1:** Chemical composition of the investigated HEAs used in the study.

Alloy Variant	Elements Content, at. %				
	Al	Cu	Co	Fe	Ni
D1	7.14	7.14	28.57	28.57	28.57
D3	13.33	6.66	26.66	26.66	26.66
D5	20.00	20.00	20.00	20.00	20.00

**Table 2:** Purity of input materials.

Chemical Element	Purity (%)
Al	99.9
Co	99.95
Cu	99.99
Fe	99.9
Ni	99.95

**Table 3:** Phase transformation temperatures obtained during DSC tests, °C.

Alloy		Heating			Cooling	
	Dissolution of Precipitates	T_S_	T_L_	Formation of Precipitates	T_S_	T_L_
D1	1253	1385	1442	1248	1361	1415
D3	1253	1335	1411	1247	1317	1390
D5	1248	1129	1340	1230	1187	1322

**Table 4:** Chemical composition of the selected phases (regions) in the D5 alloy, %at.

Element Phase	Al	Co	Cu	Fe	Ni
Bright	12.3 (±0.3)	21.9 (±0.1)	23.7 (±0.3)	23.5 (±0.1)	18.5 (±0.1)
Dark	23.5 (±0.2)	22.4 (±0.1)	10.6 (±0.1)	19.4(±0.2)	24.1 (±0.1)

**Table 5:** Mechanical properties of D1, D3 and D5 alloys.

Alloy	R_p0.2_, MPa	R_m_, MPa	E, GPa	A, %
D1	171.0 (±30.0)	369.0 (±24.9)	184.0 (±1.4)	49.6 (±11.9)
D3	271.7 (±13.8)	465.7 (±17.6)	120.6 (±8.7)	60.6 (±6.1)
D5	25.1 (±10.2)	27.2 (±9.4)	28.8 (±8.4)	0.1 (±0.03)

**Table 6:** Impact test results of the D1, D3 and D5 alloys.

Alloy Variant	D1	D3	D5
Impact energy, [J]	172.7 (±14.1)	151.3 (±11.8)	1.5 (±0.1)
Impact toughness, [J/cm^2^]	215.9 (±31.7)	189.1 (±22.5)	1.9 (±0.1)

## Data Availability

The original contributions presented in this study are included in the article. Further inquiries can be directed to the corresponding author.
